# Feasibility and effectiveness of a clinical model of sleep education for children with IDD: a record review

**DOI:** 10.3389/frsle.2026.1937989

**Published:** 2026-09-09

**Authors:** Beth A. Malow, Kasey A. Fitzpatrick, Whitney A. Loring

**Affiliations:** Department of Neurology, Vanderbilt University Medical Center, Nashville, TN, United States

**Keywords:** community, insomnia, intellectual and developmental disabilities, sleep, sleep education

## Abstract

**Introduction:**

This study was undertaken to evaluate the effectiveness and feasibility of implementing a brief community-based sleep education intervention within a collaborative clinical in academic medical setting for children with intellectual and developmental disabilities.

**Methods:**

Record reviews were conducted from a behavioral sleep clinic serving children with IDD (age ≤ 18 years). Sixty-five children and caregiver received individualized insomnia recommendations. A follow-up visit occurred 1–3 months later. Clinical notes were evaluated using a modified Pediatric Sleep and Autism Clinical Global Impressions Scale to assess effectiveness and feasibility.

**Results:**

At first follow-up, 85% of caregivers reported compliance with recommendations; 85% of whom reported significant sleep improvement. Only 20% of non-compliant families reported improvement. Compliance at first follow-up was significantly associated with improvement in insomnia (Pearson chi-square and Fisher's exact test *p* < 0.0001).

**Conclusion:**

Brief collaborative sleep education interventions are feasible and effective for improving insomnia in most children with IDD.

## 1 Introduction

Individuals with intellectual and developmental disabilities (IDD) experience higher rates of sleep difficulties than the general population (Esbensen and Schwichtenberg, 2016). Additionally, sleep problems are associated with poorer cognitive outcomes for individuals with IDD (Harvey and Kennedy, 2002). Sleep plays a vital role in enhancing brain plasticity and affects developing brain structures (Bernier et al., 2010). Sleep problems during infancy may potentially contribute to poorer executive functioning later in life (Bernier et al., 2010). Sleep also significantly affects emotional regulation in young children with IDD (Favole et al., 2023).

The heterogeneity of the underlying causes of IDD, along with the associated co-occurring conditions, complicate the diagnosis and management of sleep disorders in this population (Korb et al., 2023). For example, in autism spectrum disorder (ASD), there are often multifactorial causes affecting sleep. These include medical and psychiatric conditions, medications, and sleep habits, in addition to intrinsic features of ASD, such as overarousal (Maxwell-Horn and Malow, 2017). Pediatric insomnia is typically marked by repeated episodes of difficulty initiating or maintaining sleep, or premature wakings that lead to poor-quality sleep (Malow et al., 2012). Insomnia is seen at higher rates for children with IDD (Korb et al., 2023).

The success of pharmacological and non-pharmacological management of sleep disorders in adults and children with IDDs has not been well established. Yet, pharmacological treatments for sleep in IDD, particularly for children with ASD are widely implemented. Medications are often not effective or associated with adverse effects (Reynolds and Malow, 2011). In contrast, sleep education focused on behavioral interventions is not associated with adverse effects and is often effective (Maxwell-Horn and Malow, 2017; Loring et al., 2016; Johnson et al., 2013, 2023). Research into the use of behavioral interventions for sleep issues in children with ASD has primarily focused on parent education with young children (Vriend et al., 2011) and interventions that train parents individually or in groups (Loring et al., 2016; Malow et al., 2014; MacDonald et al., 2021). These studies established improvement in sleep onset latency, prolonged night wakings, and sleep efficiency. In some cases, improvement in children's daytime behaviors including inattention, hyperactivity, and daytime sleepiness was also noted. A systematic review of parent training incorporated into behavioral sleep interventions in ASD and IDD showed that this training was generally effective and valued by parents (Kirkpatrick et al., 2019).

Common components across most behavioral interventions include an emphasis on sleep hygiene and interventions that specifically impact lifestyle, environment, and the child's circumstances prior to sleep (Loring and Malow, 2022). The main goals of integrating sleep hygiene into treatment are to positively affect the sleep-wake cycle, establish environmental cues that create a contrast between daytime and nighttime, create a sleep conducive environment, and set expectations for sleep (Loring and Malow, 2022). Tailoring to the unique needs of the patient population with IDD is important in utilizing these treatment components. For example, adapting these recommendations for individuals with ASD by using visual supports and reinforcement systems are often essential for successful implementation, as well as identifying smaller changes that may work toward the ideal recommendations (Loring and Malow, 2022).

Given the multifactorial nature of sleep concerns in the population with IDD, there is ongoing evidence supporting the importance of utilizing behaviorally based approaches in addressing sleep problems (Loring and Malow, 2022). Using these known effective behavioral components, we implemented these behavioral interventions through a brief community-based sleep education program for parents of children with ASD. This was done through a consultative caregiver training model, which improved key components of sleep and proved to be a feasible model for both providers and caregivers (MacDonald et al., 2021). Given the prevalence of sleep disorders in children with IDD and the common barriers to accessing care for this population, there is a continued need to identify and evaluate sleep education models that realistically and effectively reach this population in a specialized way. Therefore, the aim of this study is to further examine the clinical implementation of this brief community-based sleep education intervention in the context of a collaborative clinical sleep medicine model in an academic medical setting. We performed a record review, and hypothesized that improved clinical outcomes would be associated with caregiver compliance with recommendations.

## 2 Materials and methods

### 2.1 Participants

Record review was conducted for 65 patients with a clinical history of IDD who were referred to a psychologist trained in community-based sleep education (MacDonald et al., 2021) for individuals with IDDs. The psychologist (WAL) developed the Vanderbilt University Medical Center (VUMC)'s Behavioral Sleep Medicine Clinic and had 13 years experience developing and conducting training, consultation, and coaching for caregivers, educators, and providers regarding evidence-based assessment and intervention strategies for individuals with autism spectrum disorders and related disabilities. Patients were seen at the VUMC Behavioral Sleep Medicine Clinic in person or on telehealth between June 2018 and January 2023 with data collection conducted between October 2020 and August 2023. All children were between the ages of 20 months to 18 years old with the mean age being 5.8 years. Twenty-two (34%) were 20 months to 3 years old, twenty-two (34%) were between 4 and 6 years, fourteen (21%) were between 7 and 11 years, and seven (11%) were 12 to 18 years. Further breakdown of demographics is provided in the Results section. The referral was made after being seen by a developmental medicine clinician or sleep medicine provider within the same institution. Applicable referring providers were given guidance by a sleep specialist with expertise in IDDs and the psychologist regarding who was a good fit for the clinic. Specifically, the patient must have a diagnosed IDD, and they and/or the caregiver must be open to behavioral interventions when asked in the medical appointment. Referring medical providers were also asked to first exclude or address medical reasons for sleep concerns (e.g., obstructive sleep apnea, gastroesophageal reflux, seizures) either prior to or in conjunction with the behavioral referral. The referral could be made while these concerns were still being assessed or addressed (e.g., while waiting for a sleep study) if the need for behavioral intervention in conjunction with the medical concerns seemed warranted based on the medical provider's evaluation. The psychologist communicated back to the referring medical provider to provide relevant updates while the medical provider continued to care for the child, including sharing progress or medical questions the patient may have.

Referral concerns consisted of difficulty falling asleep and/or night wakings. Race/ethnicity, age, diagnosis, psychotropic medications, and sleep-related surgical interventions were collected.

Patients and caregivers typically met with the psychologist for 1 to 3 months (average 2.5 months) after the initial visit to report their successes or challenges in implementing the initial recommendations. They also shared sleep improvements or changes and any questions/concerns regarding the behavioral recommendations. Overall, caregivers reported being motivated to provide realistic information and barriers to implementation in order to receive support from the psychologist, thus minimizing the risk of presenting information inaccurately or favorably. Depending on the child and caregiver's needs, additional follow-up visits were typically scheduled 1 to 6 months afterward if warranted. Following the sleep education intervention, the psychologist communicated back to the referring clinician the progress made, or lack thereof, for the clinician's continued management. The psychologist could be consulted again if new sleep concerns developed or if the initial plan was no longer effective. Data was collected and reviewed through a secure data collection system (Research Electronic Data Capture- REDCap) and approved by our Institutional Review Board.

### 2.2 Sleep education

Based on our previously published curriculum (Malow et al., 2014; MacDonald et al., 2021; Loring et al., 2016), the sleep education intervention consisted first of a 90-min in-person or telehealth appointment with the psychologist to review the child's clinical diagnosis(es) and pertinent history. The psychologist conducted a behavioral sleep assessment where the caregiver (and child when able) was asked to provide details regarding the child's sleep profile, daily routines, and previous and current sleep interventions. The option for a telehealth model was initiated during the COVID-19 contact restrictions. Once in-person appointments were reinstated, caregivers were given the option for an in-person or telehealth appointment based on their preference. Caregivers could choose solely telehealth or in-person appointments or a combination based on their needs. Telehealth overall became the preferred option with 43 out of 65 parents preferring telehealth for appointments due to convenience in scheduling and distance necessary to travel for in-person appointments for many patients.

During the remainder of the initial appointment, the psychologist provided individualized recommendations drawn from 12 possible categories, tailored to the specific information gathered during the sleep assessment. This individualized approach aimed to align the strategies with the unique needs of the child and family, rather than relying on a generic list of behavioral sleep recommendations. Specifically, caregivers reported that, in their past experiences, such recommendations were often delivered in brief appointments, typically as simplified handouts or generalized advice, with little attention to how they might be realistically implemented for a child with IDD. As a result, caregivers often felt frustrated and concerned that the provider did not understand the unique needs of their child and family and were left to attempt strategies that were impractical or unworkable without guidance on how to adapt them from the “ideal” model.

In contrast, our approach was intentionally collaborative. A significant portion of the visit was dedicated to working with the caregiver to evaluate the feasibility of each recommendation and to determine how it could be specifically implemented within their family context. For example, for a child who had limited physical activity, the psychologist and caregiver brainstormed together to determine specific activities that would realistically promote meaningful movement/exercise within the child's current routines. They also identified together what additional supports may be helpful to aid in the implementation of the recommendations identified (e.g., a visual timer during exercise time, a morning reward system for staying in bed all night).

Additional strategies were recommended to uniquely support children with IDD based on evidence-based practices that promote learning, such as visual supports, reward systems, and self-monitoring tools. The caregivers were asked to immediately begin implementing recommendations. Sixty-minute follow-up in-person or telehealth appointments were scheduled to review implementation of recommendations and assess sleep changes. The focus of these follow-up appointments was to troubleshoot issues that may have arisen while implementing the recommendations and to adjust or add recommendations based on reported sleep changes. Table 1 provides detail for each general recommendation type with context regarding why they are important to sleep hygiene and this population (Loring and Malow, 2022).

**Table 1:** Categories of sleep recommendations.

Recommendation #	Content of Recommendation
Recommendation 1	**Morning routine:** Establishing a consistent morning routine that clearly distinguishes between nighttime and daytime helps prevent or reduce the likelihood of the child returning to sleep. This routine should start with leaving the sleep environment promptly upon waking and changing out of sleep clothes. It should then include a series of activities performed in the same sequence and with the same timing every morning, whether it is a school day or a non-school day and should ideally include exposure to light and some form of movement.
Recommendation 2	**Daytime light:** Exposure to daytime light regulates the sleep-wake cycle and distinguishes between daytime and nighttime. Natural light is optimal, but if unavailable, supplementing artificial light such as overhead lights or additional lamps can also be effective.
Recommendation 3	**Bedroom use:** A key aspect of sleep hygiene is establishing a clear association between sleep and the bedroom. All stimulating activities (e.g., playing, watching TV) should occur outside of the bedroom to avoid associating the sleep environment with other activities. If it is not feasible to completely remove certain activities from the bedroom, reducing the overall time and frequency of stimulating or non-preferred activities in the sleep environment can still be beneficial.
Recommendation 4	**Exercise:** Consistent, purposeful exercise during daytime hours promotes good sleep. Exercising too close to bedtime can lead to overstimulation that disrupts sleep. Whenever feasible, it is beneficial for exercise to take place outdoors or near windows to combine physical activity with exposure to natural light. For children with self-regulation differences and/or high activity levels, it can also be helpful to identify exercise opportunities with structure embedded and with more sedentary/calming activities before and/or after the activities.
Recommendation 5	**Caffeine:** Avoiding food and drinks containing caffeine reduces overstimulation at night. Caffeine may linger in the system longer than noticeably externally, and individuals vary in their sensitivity to it. If eliminating caffeine entirely is not immediately feasible, strategies such as diluting caffeinated drinks with water, switching to caffeine-free versions, or establishing a cut-off time after which caffeine is no longer available to the child can be helpful steps toward this goal.
Recommendation 6	**Naps:** While daytime naps are developmentally appropriate at certain ages, they can disrupt nighttime sleep if they are no longer necessary, occur inconsistently, or happen late in the day. For developmentally appropriate naps, focus on consistent length and timing each day and incorporating similar routines and environment that are present at bedtime. If there are specific times of day or activities when unintended naps typically occur, engage in alternatives to avoid the urge to nap (e.g., playing interactive games in the car, outdoor activities after school instead of watching TV). If naps are taken, try to keep them as short as possible (e.g., less than 45 min per day) and avoid napping too late in the day (e.g., no later than 4 pm).
Recommendation 7	**Bedtime routine:** In the 15–30 min before bedtime, establish a consistent bedtime routine featuring simple, enjoyable, and relaxing activities performed in the same sequence each night. This routine acts as a precursor strategy, signaling that bedtime is approaching, offering predictability, and calming the mind and body before sleep. Stimulating, challenging, or less preferred activities should ideally be scheduled early in the routine or removed from the bedtime routine and completed earlier.
Recommendation 8	**Bedroom environment:** Consistency in the sleep environment is sleep-promoting. For individuals with IDD who are sensitive to sensory stimuli, it is important to maintain visual and tactile stimuli that minimize distraction, overstimulation, or changes during the night. Ideally, the sleep environment should be dark without additional lights or sounds when falling asleep. However, gentle background noise may be necessary to mask household sounds, soothe the child, or signal nighttime. Since blue light from electronic devices suppresses melatonin production and screen activities can be stimulating, it is beneficial to aim for turning off all screens (e.g., phone, TV, computer, tablet) at least an hour before bedtime.
Recommendation 9	**Brief and boring approach:** After it has been communicated to the child that it is bedtime, the caregiver should begin making their interactions “brief and boring” to help child understand that there is no benefit of additional attention by staying up longer or waking up in the night. The best response should be to guide the child back to bed rather than talking, singing, cuddling, or other activities that may stimulate the child.
Recommendation 10	**Co-fading techniques:** These specific practices focus on gradually fading out co-sleeping of children with caregivers. These can be customizable depending on the severity. Examples include caregivers sitting on the bed in the room at bedtime rather than laying down or touching the child in any way or putting a mattress on the floor in the caregiver's bedroom to encourage sleep outside of the caregiver's bed. The goal is to gradually work toward the child to sleep in their own bed for the entirety of the night.
Recommendation 11	**Visual schedule (visual supports):** A visual schedule (visual supports) uses pictures and symbols to help a child understand what will take place that day. This can bring predictability and help with the transition from one activity to another. These schedules and supports can be customizable and used as a cue to distinguish daytime from nighttime as well as communicate expectations regarding the implementation of other recommendations provided (e.g., a “Stop” sign to indicate when bedroom is off limits, a visual schedule of the morning and bedtime routines, a choice board for exercise options).
Recommendation 12	**Other recommendations:** These recommendations did not fall in the categories above. Examples included advising families to watch sleep curricula online or reading specific books to alleviate child's anxiety around bedtime.

### 2.3 Outcome measures

#### 2.3.1 Recommendation and implementation scores

To quantify reported implementation of the specific categories of recommendations given, a recommendation score and an implementation score were calculated for each patient using a coding process from parent reports following the completion of at least an initial and follow-up session. This assessment was done by a research coordinator with expertise in chart review. The psychologist was masked to the following ratings and outcome at the time of the appointments and was not involved in the scoring via chart review.

Not every patient received all 12 categories of recommendations but instead received the combination of recommendations deemed appropriate by the psychologist based on the sleep behavioral assessment conducted at intake. Each category of recommendation given at the initial appointment was assigned a 1. Any category of recommendation not given/not specified/not applicable was assigned a 0. The total recommendation score was determined by adding all the categories of recommendations together.

Additionally, upon review, the overall implementation of recommendations was given a total implementation score by the research coordinator after the appointment date retrospectively based on the one psychologist's assessment of the caregiver report at the follow-up appointments. (2 = Yes, caregiver/patient complied with implementing recommendations; 1= Partial/Incomplete compliance; 0 = No, they did not comply/Not specified/Not applicable). The implementation score was determined by adding all compliance numbers for each category of recommendation together. For a successful compliance/implementation rating, the participant must have an implementation score equal to or greater than their recommendation score. This indicates that they complied with half or more of the categories of recommendations successfully. For example, if a patient is given 8 out of the 12 categories of recommendations, they are given a total recommendation score of 8. If they complied with 4 of the categories of recommendations, the total implementation score would be 8. This coding process is demonstrated in the case sample in Supplementary Table 1.

#### 2.3.2 Pediatric sleep and autism clinical global impressions scale (pediatric sleep CGI)

Charts were analyzed by using an adapted form of the Pediatric Sleep and Autism Clinical Global Impressions Scale (Malow et al., 2016). This scale (Supplementary Table 2) was modified to evaluate and rate the level of severity of sleep onset latency and night wakings through caregiver-reported clinic notes. Each child's chart was labeled by coordinator with a number 1 through 7 reflecting either increased levels of sleep onset latency or night wakings or depending on the referral concern. This ranking was used to compare changes at each visit.

Average sleep latency, along with the number of nights a week child had night wakings, and how many times a night the child awoke, by caregiver report, were also collected at the initial and follow-up appointments. The psychologist had a standard approach for reporting improvement in the chart, which included specific changes reported by the parent in sleep onset latency and frequency and length of night wakings and the significance of these changes. These were the statements used by the coordinator to determine the appropriate rating. The research coordinator reviewed each chart and used The Pediatric Sleep and Autism Clinical Global Impressions Scale (Pediatric Sleep CGI) to classify the child as: Improved, No change, or Worse. Masked double coding was conducted by an independent study coordinator on 15% of randomly chosen participant charts. There was an 86% agreement found on CGI ratings at first and second follow-up as well as the overall rated improvement.

The demographics (e.g., age, sex) and other relevant information (e.g., IDD diagnosis, medications) of the children enrolled in the project were collected from chart review. Graphs were created to demonstrate changes in sleep onset delay and night wakings before and after sleep education and a related-samples Wilcoxon signed-rank test was performed. A Pearson chi-square test and Fisher's exact test were used to determine the relationship between compliance at first follow-up and change in insomnia, combining reported improvement into one category and no change and worse insomnia into a second category. Statistical analysis was performed using SPSS and R software.

## 3 Results

### 3.1 Study population

All children were between the ages of 20 months to 18 years old with the mean age being 5.8 years old. Forty-two (64%) were male and twenty-three (36%) were female. Our sex distribution reflects the male to female representation for children diagnosed with IDD in the United States with 67% of children with IDD as male and 33% as female (National Core Indicators, 2020). Fifty (77%) of children were White, six (9%) were Black/African American, and the other nine children (14%) were labeled as biracial, multiracial, or unknown. Fifty-eight (89%) children were listed as Not Hispanic/Latino, or Spanish, four (6%) were listed as “Other Hispanic/Latino, or Spanish,” and three (5%) were listed as “Unknown/Not Available.”

The most common diagnosis was ASD with 49 (75%) children having this diagnosis in their medical charts. Additionally, 28 (43%) had developmental, global, or speech delay, 18 (28%) had attention deficit hyperactivity disorder (ADHD), and one (1.5%) had Trisomy 21. Most of these patients had more than one of these diagnoses. For example, 14 (22%) participants had both ASD and ADHD. In addition, some of these patients may have had additional medical or psychiatric diagnoses that were not tracked. For the purpose of this report, the IDD diagnosis(es) was the primary focus of diagnostic tracking.

Patients and caregivers typically met with the psychologist 1 to 3 months (average 2.5 months) after the initial visit to report their successes or challenges in implementing the initial recommendations. Nineteen (29%) children had their first appointment prior to the COVID-19 pandemic (all in-person). For those who were seen during or after the COVID 19 pandemic, they were given an option to do an in-person or telehealth visit. Four (9%) did in-person and 42 (91%) did telehealth visits. Telehealth became the preferred option for appointments due to convenience and length of the appointment. At the initial visit, 37 (57%) children were taking prescribed psychotropic drugs (e.g., clonidine, guanfacine, gabapentin, amphetamine and dextroamphetamine), and 29 (45%) were documented as taking melatonin.

### 3.2 Pediatric sleep CGI ratings

**Figure 1:**
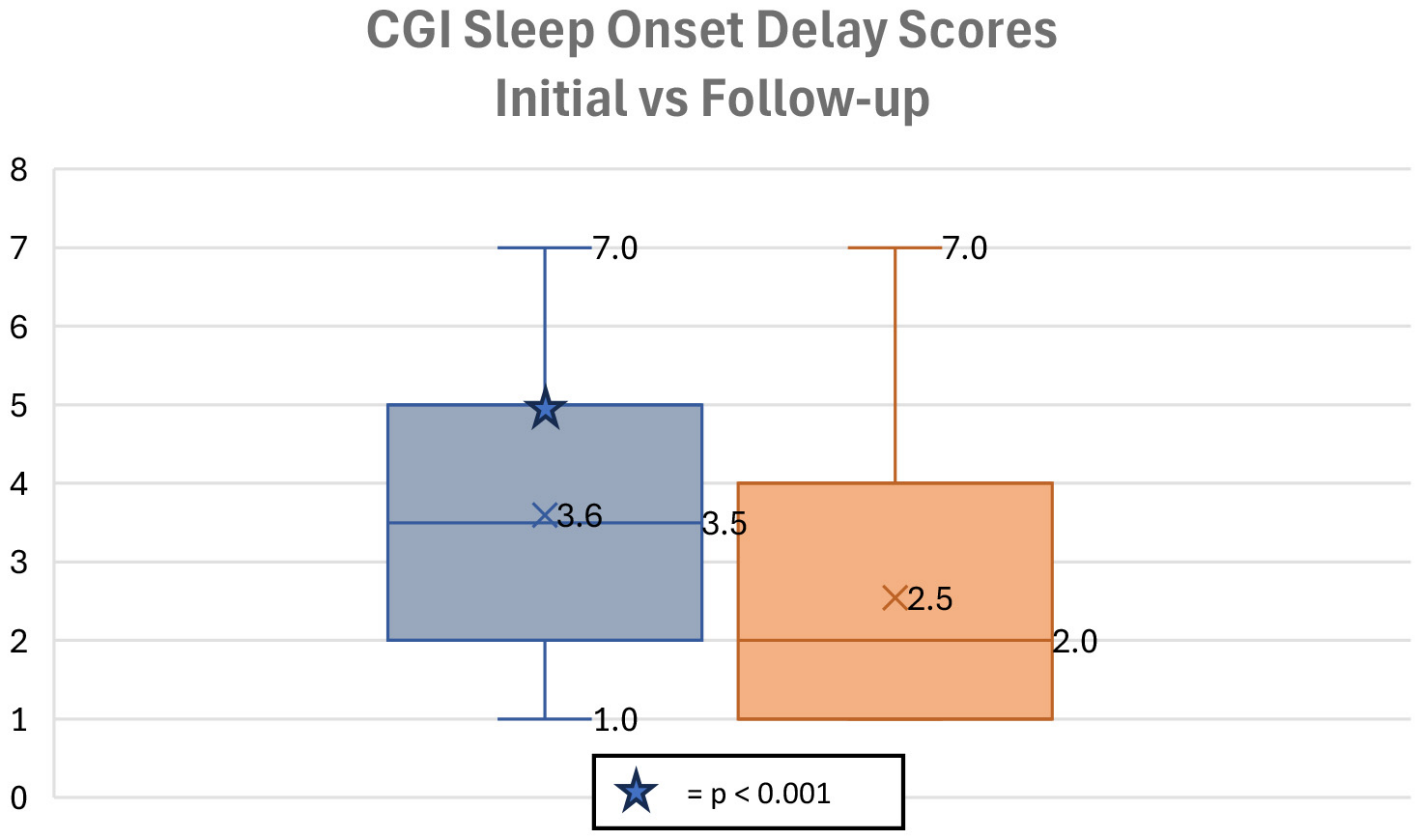
The Pediatric Sleep and Autism Clinical Global Impressions Scale (Pediatric CGI) category of sleep onset delay (SOD) at baseline and the first follow-up is depicted. This box and whisker graph presents the mean (noted to right of the “x”), median (noted by the horizontal line within the box), quartiles (noted by the horizontal line at the top and bottom of the box), and range (noted by the horizontal lines outside the box). There was a statistically significant improvement in sleep onset delay (*p* < 0.001).

**Figure 2:**
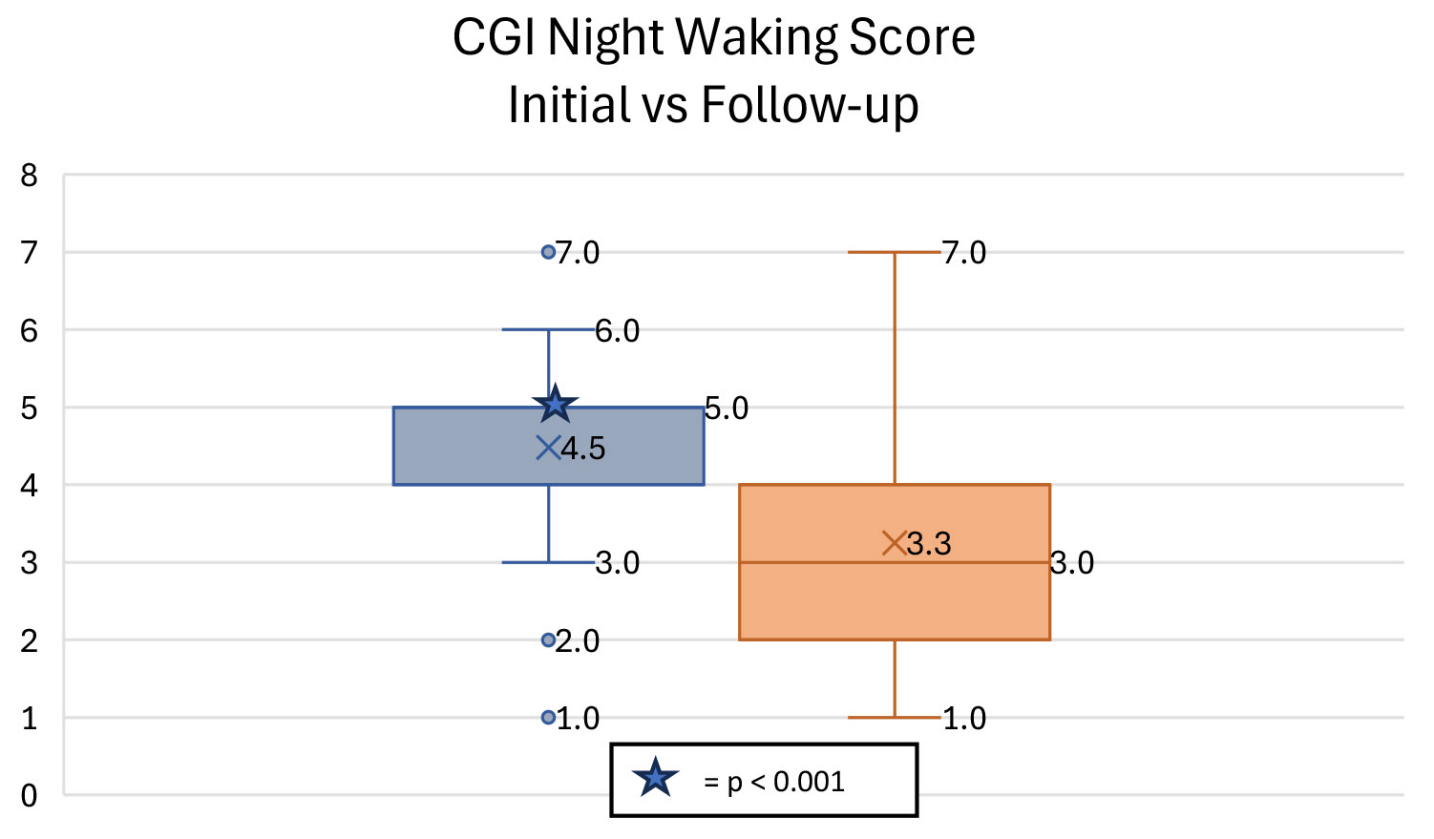
The Pediatric Sleep and Autism Clinical Global Impressions Scale (Pediatric CGI) category of night wakings at baseline and the first follow-up is depicted. This box and whisker graph presents the mean (noted to right of the “x”), median (noted by the horizontal line within the box), quartiles (noted by the horizontal line at the top and bottom of the box), range (noted by the horizontal lines outside the box), and outliers (noted by the dots). There was a statistically significant improvement in night wakings (^⋆^*p* < 0.001).

Difficulty falling asleep was reported by 47 (72%) of caregivers and night wakings by 57 (86%) of caregivers. Difficulty falling asleep and night waking together was reported by 40(61%) of caregivers. The Pediatric sleep CGI categories of sleep onset delay and night wakings at baseline and the first follow-up are presented in Figures 1, 2. With follow-up, there were statistically significant improvements in both sleep onset delay and night wakings. Median SOD CGI score went from 3.5 to 2 (initial to follow-up). Median Night Wakings CGI score went from 5 to 3 (initial to follow-up)

### 3.3 Implementation measures

As shown in Table 2, 55 (85%) caregivers complied with more than half of the recommendations given by the first follow-up. Of these caregivers, 47 (85%) reported an improvement in the child's insomnia, six (11%) reported no change, and two (3%) reported worse insomnia. In contrast, in the 15% of caregivers who did not reach this level of compliance, two (20%) reported improvement, six (60%) reported no change, and two reported worse (20%) insomnia. Compliance at first follow-up was significantly associated with improvement in insomnia (Pearson chi-square with Yates correction = 16.2, *df* = 1, *p* < 0.0001; Fisher's exact test: Odds Ratio = 21.8 (95% confidence interval = 3.5, 249.7; *p* < 0.0001). The Yates correction and the Fisher's exact test were used because of the small sample size in the reported improvement/did not comply cell. Of those who had a second follow-up appointment, 21 (91%) complied with more than half of the recommendations.

**Table 2:** Reported compliance and outcome at first follow-up.

Outcome in sleep at first follow-up				
Compliance outcome at first follow-up	Total	Reported improvement	Reported no change	Reported worse
Complied with more than half of recommendations	55 (85%)	47 (85%)^*^	6 (11%)	2 (4%)
Did not comply with more than half of recommendations	10 (15%)	2 (20%)	6 (60%)	2 (20%)
Total	65			

^*^Compliance at first follow-up was significantly associated with improvement in insomnia.

For the analysis, reported no change and reported worse were combined into one category. Eight participants (15%) complied with more than half of recommendations and did not improve, and 8 participants (80%) did not comply with more than half of recommendations and did not improve.

Compliance at first follow-up was significantly associated with improvement in insomnia (Pearson chi-square with Yates correction = 16.2, df = 1, *p* < 0.0001; Fisher's exact test: Odds Ratio = 21.8 (95% confidence interval = 3.5, 249.7; *p* < 0.0001). The Yates correction and the Fisher's exact test were used because of the small sample size in the reported improvement/did not comply cell.

The most common recommendations given were to establish a morning routine, increase exposure to daytime light, increase daytime exercise, and establish a bedtime routine. Establishing a morning routine, increasing daytime exercise, and establishing a bedtime routine were the most successfully implemented recommendations reported by families.

Seventeen caregivers reported that their child started a new pharmacological intervention drug and/or having surgery after the initial appointment (e.g., tonsillectomy/adenoidectomy, pharmaceutical drugs affecting sleep). Ten caregivers reported new pharmacological interventions, three caregivers reported surgery after the initial appointment, and four caregivers had both surgery and pharmacological interventions after the initial appointment. In those children who had surgery and/or pharmacological intervention after the initial appointment, 15 (94%) of their caregivers complied with recommendations. Of those caregivers whose children had surgery and/or pharmacological intervention and complied with recommendations, 13(87%) reported improvement, two reported no change (13%), and two (13%) reported worse sleep by first follow-up.

## 4 Discussion

In this report, we summarize a model for clinical implementation of a brief community-based sleep education intervention for children with ASD. This intervention is implemented in the context of a collaborative sleep medicine model that extends to the IDD pediatric population. Rather than evaluating efficacy through large randomized controlled trials, the primary aim was to assess the feasibility and utility of this intervention within an existing academic medical care structure. By sharing this model, we hope to inform future research, including randomized and comparative effectiveness studies, and to provide a replicable approach for settings with limited access to specialized sleep services.

Many existing sleep education programs are lengthy and place unrealistic demands on caregivers of children with IDD. Additionally, these programs are often delivered by independent practitioners without integrated collaboration with medical providers. In contrast, our model highlights a feasible, brief intervention that demonstrates comparable sleep improvements while remaining accessible within routine sleep medicine care. This integrated approach allows for continuous coordination of medical and behavioral insomnia management, which may be more realistic for families navigating care across providers. Notably, families frequently reported meaningful improvements following a single appointment.

A variety of parent-focused sleep education strategies have been studied, including pamphlets alone (Adkins et al., 2012) and combinations of pamphlets, tip sheets, and videos (Malow et al., 2021). While these approaches were perceived as helpful, parents consistently reported a need for coaching from a trained educator to successfully implement recommendations. In response, we adopted a coaching-based sleep education model that emphasizes individualized strategies tailored to each child and family. Initially implemented in academic centers through individual and group formats (Reed et al., 2009; Malow et al., 2014) and later expanded to community settings (MacDonald et al., 2021), this model is further supported by our findings, which suggest that families of children with IDD benefit most from guided, personalized intervention

The effectiveness of this approach likely reflects its emphasis on feasibility and collaboration. While many sleep hygiene recommendations are widely known, they are often shared in ways that fail to propose realistic adjustments. Caregivers often attend appointments overwhelmed and sleep-deprived, making generic recommendations difficult to translate into action. Families struggle to implement these recommendations in a manner that fits their current schedule and capabilities. Furthermore, uniform recommendations may also erode trust when families feel their child's unique needs are not fully understood. This model allows providers to collaborate with caregivers, considering their circumstances, and develop realistic goals and timelines with appropriate support, acknowledging that “one size does not fit all.” The collaborative and highly individualized nature of this model likely contributed to the strong implementation and improvement rates.

Another crucial aspect contributing to the success of this model is its utilization of an interdisciplinary team of clinicians to enhance patient care. It emphasizes a collaborative approach with referring clinicians, which goes beyond a straightforward referral to a behavioral sleep clinic. Effective communication with referring clinicians and ongoing monitoring significantly improves patient care and outcomes, fostering a collective effort to enhance sleep. Additionally, clinicians maintain communication between appointments and collaborate on treatments, providing ongoing support to patients while awaiting follow-up details such as bloodwork or sleep studies. Our report also emphasizes how medications and surgical interventions are not always quick fixes but are often more effective when combined with personalized sleep recommendations.

This review adds to evidence that behavioral sleep interventions across formats such as group training, in-person consultation, telehealth, and community-based models are most effective when they emphasize caregiver coaching, individualized planning, and realistic adaptation to family routines. The comparable outcomes observed in our brief model suggest that shorter, integrated clinical approaches may serve as a viable and accessible alternative to more intensive behavioral programs, expanding options for families who may be unable to participate in lengthy or multi-session interventions.

### 4.1 Limitations

Our work has several limitations. First, our outcome ascertainment was subject to bias as we were conducting this chart review (non-randomized) study in the context of clinical care. The psychologist who provided care also reported improvement or lack of improvement, and it is possible that bias in the direction of more improvement may have been present. To mitigate this potential bias, we ensured that the psychologist had a consistent process for extracting parent reports and determining an overall clinical impression of changes in sleep across all patients. Furthermore, when the psychologist began documenting care, she was unaware that a retrospective chart review would occur at a later date. Therefore, she documented based on best ethnical practice of noting improvement, lack of change, or worsening of symptoms as part of their medical record.

We also performed masked coding, by an independent study coordinator unaware of the other coder's results, on 15% of randomly chosen participant charts. We recognize that both coders were aware that all patients had received an intervention given the record review format of this study. To mitigate this potential bias, coders used criteria based on standardized and broadly accepted measures from the existing sleep literature (e.g., sleep onset latency, frequency and length of night wakings) incorporated into the Pediatric Sleep CGI. Although reliance on a single clinician's assessment introduces potential bias, the psychologist assessed reported implementation and sleep patterns through a variety of means throughout the visits and provided summative impressions of these based on the interaction as a whole, rather than brief screening tools alone with yes/no options. These details were crucial for a comprehensive review though we also recognize that there may be inconsistencies that affect the precision of ratings. Second, outcomes and implementation were assessed via caregiver report rather than directly from children through objective measures such as actigraphy or polysomnography. However, caregivers appeared motivated to provide accurate information to receive targeted support, thus minimizing the risk of presenting information inaccurately or favorably. Furthermore, the Pediatric Sleep CGI, while useful for clinical impressions, does not capture the full range of sleep parameters that standardized objective tools such as polysomnography or actigraphy might provide. Yet, both have limitations related to tolerance of sensors in IDD populations and increase family burden in assessing sleep patterns.

This is intentionally a small-scale clinically based review designed to evaluate the realistic implementation of time-limited sleep education models in a collaborative academic medical setting and share this model with other professionals. The size of the current data set does not allow for statistical analysis of factors including the impact of telehealth vs. in-person structures, surgical/pharmacological intervention, and the association between specific IDD diagnosis and outcome. This model was implemented as part of routine clinical care rather than as a controlled research protocol, which we also acknowledge can have inherent variability in documentation, caregiver descriptions, and follow-up timing.

Our review did not systematically track several factors that may meaningfully influence outcomes, such as caregiver stress, socioeconomic barriers, family resources, or differential access to support services. Prior research has shown that these contextual variables can impact both the implementation of sleep recommendations and the sustainability of behavior change. Similarly, although medication and surgical interventions were documented, their specific interactions with behavioral recommendations were not fully analyzed and may represent confounding influences. All of these factors limit generalizability of our work.

Future research could explore a comparative study between online resources and consultative models, considering if different populations require varying levels of intervention. Exploring this intervention model with larger populations of children with IDD, and specific IDD populations, such as Trisomy 21 or ASD, could provide insights into unique needs. Factor analytic studies would also be useful in examining which commonly recommended intervention strategies are most impactful for the IDD population, as well as certain age ranges or diagnostic groups. Furthermore, developing validated tools for screening and assessing sleep disorders in IDD populations would be beneficial.

As sleep problems continue to be an area of concern for many children with IDDs, it is important to consider sleep education as an effective tool for families. Despite being a time-limited consultative model, our findings showed very high levels of implementation, noted improvements in sleep behavior, and indicated a relationship between implementation and improvement.

This model proved feasible for both caregivers and providers, demonstrating a structure that integrates researched models into collaborative multidisciplinary settings to serve a complex patient population. Key factors contributing to its success included a well-defined referral pathway, ongoing communication with medical sleep providers, and highly individualized recommendations based on family profiles, utilizing evidence-based practices known to be effective in this population overall. Given that sleep problems remain a significant concern for many children with IDDs, emphasizing brief consultative sleep education models as an effective tool for families is crucial.

## Data Availability

The raw data supporting the conclusions of this article will be made available by the authors, without undue reservation.
