# Supplementary material for: Feasibility and effectiveness of a clinical model of sleep education for children with IDD: a record review
Source: Front Sleep. 2026 Sep 9;5:1937989. doi: 10.3389/frsle.2026.1937989 (PMC13597379; doi:10.3389/frsle.2026.1937989)
Supplement: Supplementary file 1 [file Supplementary_file_1.docx]

**Supplemental**

*Deidentified case along with how it was coded.*

George is 4-year-old male on the autism spectrum with developmental delay and a diagnosis of insomnia. He lives with his mother, father, and two siblings. George presented with difficulty initiating sleep, frequent night wakings, and associated daytime irritability and challenging behaviors. His current bedtime is 9:00 pm, but it will often take him 3-4 hours to fall asleep, usually around 12:00 or 1:00 am. He sleeps in his parents’ room in a separate bed but sometimes falls asleep in their bed or gets in their bed during the night. Parents report he will often wake up during the night and sometimes go to his siblings’ rooms to watch TV. He typically falls back to sleep quickly but he is sometimes up until they get him up for the day. He was previously seen by a medical sleep provider and prescribed gabapentin and melatonin to help with presenting insomnia. However, parents report struggling with the dosage and timing of his medications. He is often sleepy and irritable in the morning throughout his morning routine activities with the addition of these medications. He is also still having sleep onset delay and night wakings despite medication use.

When assessing the current state of sleep hygiene and daytime behaviors associated with sleep, his parents report that he attends a special education preschool program five days a week where he also receives speech and occupational therapy. On school days, parents get him up around 7:00 am. He is sometimes already awake and other times sleeping and very difficult to wake. They then get him washed up, put his clothes on, pack up, and get him on the bus. He is at school until 3:30 pm. There are scheduled naps at his school at 10:00 am. He initially slept during these scheduled naps, but this has been variable following medication changes, so the consistency and length of the naps is unclear. He sometimes falls asleep on the bus for an approximately 30-minute nap but does not nap at home. His parents and the school report an increase in challenging behavior on the days when his sleep has been poor the night before. On weekends, he wakes up, on his own, by 8:00 or 9:00 am and plays with his tablet or watches TV. He sometimes eats or drinks caffeinated items, but these are limited. There is no outlet for purposeful exercise at this point, but he is reportedly “always on the go,” jumping, climbing, and throwing objects. He spends a large amount of time in the bedroom where he sleeps. This includes eating/drinking, watching TV, playing on his tablet, playing in the bed, and jumping on the bed. In the evening, George’s family typically eats dinner around 6:00-7:00 pm. Before bed, they will have him take a bath, put on pajamas, and brush his teeth. Baths are stimulating to him, and he eventually takes off his pajamas by the time he falls asleep. There are no lights or sounds in the room at bedtime and they have blackout curtains. His siblings are sometimes still awake downstairs and may have their TVs on. His mother leaves for work at 11:00 pm, so his father puts him to sleep if he is not asleep by then. George’s father often allows George to get in bed with him to fall asleep. If George wakes up during the night, his parents try to move him back to his bed, but do not always do so.

With the given information and CGI rating scale, George’s sleep onset latency was rated as a level 6 as “severe sleep onset latency” due to it taking him more than two hours or more to fall asleep, 3-5 nights a week. He was also rated level 4 as “moderate night wakings” due to him awakening once to several times during the night and returns to sleep within 15 minutes on most occasions, with this occurring every night or almost every night.

*The recommendations and coding*

Morning Routine**:** Geroge should have an established consistent morning routine. Through discussion with parents, it was determined that they aim at getting him out of bed and out of sleep clothes promptly. Specifically, waking him, moving him downstairs for washing up and dressing, and ensuring he remains sufficiently awake throughout the routine to prevent falling back asleep. It was also recommended that he maintain consistent activities and timing of this routine on both school and non-school days and avoid delaying wake-up time by more than an hour on non-school days. It was also encouraged that his caregivers incorporate movement to discourage prolonged snoozing or lingering after waking as well as ensuring that he was in rooms with ample natural light during the morning routine (e.g., opening blinds and turning on lights).

Daytime Light**:** George should be in areas throughout the day with a lot of light, particularly natural light, when possible.

Bedroom Use**:** George should stay out of his bedroom except when it is time for bed. Specifically, it was discussed relocating activities like watching TV, rough housing, or jumping on the bed elsewhere during the day since these were particularly stimulating activities that currently occurred in the bedroom.

Exercise**:** George should receive at least 30 minutes of purposeful exercise during the daytime hours, preferably outside when possible so that it is paired with natural light. Based on the discussion during the appointment, realistic options identified were a mini-trampoline and/or dance videos immediately after school for 30 minutes on indoor days and playing outside on days when the weather is nice.

Caffeine**:** George should avoid food or drinks that have caffeine, including chocolate milk, soft drinks, and tea. Ideally, these would be eliminated altogether, but since that it was not possible initially, it was encouraged that parents consider diluting these options with water, replacing them with caffeine-free versions, identifying a time after which he cannot have them (e.g., no more after lunch), and/or identifying a limit of how much he can have of each of them each day (e.g., only 1 food or drink a day with caffeine in it).

Naps**:** Geroge should decrease the “cat naps” that he may have during the day. It was suggested to parents that someone on the bus engage in interactive activities before he falls asleep to ride out the urge to nap before the nap begins. Given the inconsistency in sleeping during the scheduled nap at school, it was recommended that they schedule his therapies during that time to help him stay awake. If this is not possible, it was suggested they ask if he would be allowed to play with quiet toys in a specified area of the room instead of trying to nap. If he does fall asleep despite these measures, try to keep the amount of napping at less than 45 minutes a day.

Evening Activities/Bedtime Routine**:** Geroge should have an established consistent evening activities and bedtime routine. It was encouraged that his parents create a calm and structured environment after dinner to prepare George’s body for bed by reducing light and sound and minimizing overly stimulating or physical activities. Based on the discussion during the appointment, it was determined that George should take a bath immediately after dinner, as it seemed to be stimulating for him, and then find a quieter area of the house where he can engage in less physically demanding activities until bedtime. If he has access to a tablet or TV during this time, it was emphasized to lower the volume and screen brightness. In the 30 minutes before bedtime, it was encouraged that parents establish a consistent bedtime routine featuring relaxing and preferred activities performed in the same sequence every night.

For stimulating, challenging, or less preferred activities, it was encouraged they incorporate them earlier in the routine. It was decided that they would have him eat a snack and brush his teeth before entering the bedroom and consider having him sleeping in only his diaper instead of pajamas, as he tends to remove them as part of bedtime resistance. It was encouraged that his caregivers sit beside him to read a book rather than lying down with him once he is in bed. Then, before he falls asleep, get up to turn off the light. Due to his current sleep onset time, it was suggested that they temporarily delay putting him to bed until around 10:30 pm. Once this pattern becomes consistent, bedtime could gradually be adjusted earlier as necessary to ensure adequate sleep by putting him to bed 15 minutes earlier every few nights as he is successfully falling asleep at that time.

Bedroom Environment**:** Geroge should have a consistent bedroom environment. It was encouraged that his caregivers continue to make sure the bedroom is dark with no additional lights on when he falls asleep (such as the hall light) that will be turned off during the night. It was also suggested they incorporate a consistent noise that comes from a white noise machine, fan, or dehumidifier to drown out other noises in the house, to calm, and to serve as an additional cue that it is bedtime. It was also offered that they consider adding a body pillow to his bed to mimic being in bed with the caregiver.

Sleep Resistance/Night Wakings**:** Geroge should have brief and boring interactions during nighttime wakings. It was encouraged that his caregivers’ interactions become “brief and boring” after it had been communicated that it was bedtime to George. If he attempts to get into bed with them, they should calmly and quickly place him back in his bed with minimal interactions. It was suggested that if he gets up before falling asleep or during the night, they guide him back to his bed as quickly as possible and use the least number of words possible while referencing the white noise machine to remind him that it is still nighttime, so he should be in bed.

George and his family were scheduled for a follow-up appointment six weeks later to assess progress and make additional treatment recommendations. Together, his caregivers and the sleep psychologist reviewed the implementation of the recommendations above and progression in the goal areas identified. Parents specified that they were able to implement the morning routine, increase daytime light, increase outdoor play/exercise, limit naps, decrease light and movement around bedtime, and limit interactions during night wakings. His parents indicated significant improvement since the last appointment. They reported that he fell asleep much more easily, typically by 8:30pm. His father was no longer laying down with him while he fell asleep. George would occasionally wake up once during the night between 2:00 and 3:00 am but was easily redirected back to sleep within 15 minutes. Parents continued to give him gabapentin about an hour before bedtime and melatonin 30 minutes before bed. They noted the significant improvements above after the behavioral recommendations were implemented in conjunction with the medication. His parents were very pleased with the recommendations and his medication schedule at the time and would be in contact for additional treatment recommendations if sleep worsened again.

Nine recommendations were made and of these nine, six were fully implemented. With these nine recommendations suggested, this patient would have a recommendation score of 9 at their first appointment. A perfect implementation score would be 18 if all recommendations were implemented. At the follow up appointment, parents specified they were able to fully implement (2 points each) a consistent morning routine, increase daytime light, increase outdoor play/exercise, limit naps, decrease light and movement around bedtime, and limit interactions during night wakings which means this patient fully implemented six of the recommendations with an implementation score of 12. They were able to fully comply with more than half of the recommendations given.

With the given information and CGI rating scale, George’s new sleep onset latency was rated as a level 1 as “borderline or barely having sleep onset delay” since he now falls asleep within 30 minutes on all nights with rare exceptions with his 8:30 pm bedtime. His new rating for night wakings is a level 2 as “borderline or barely having night wakings” since he occasionally wakes up once during the night but is easily redirected back to sleep within 15 minutes. These would both be viewed as significant improvements.

The case above illustrates a common scenario where parents find it challenging to implement recommendations that accommodate their current schedules and capabilities. However, through brainstorming and discussion at appointment, the family felt they could determine realistic goals moving forward. Additionally, this case presents how quickly families can see results after just one appointment.
