# Supplementary material for: Feasibility and effectiveness of a clinical model of sleep education for children with IDD: a record review
Source: Front Sleep. 2026 Sep 9;5:1937989. doi: 10.3389/frsle.2026.1937989 (PMC13597379; doi:10.3389/frsle.2026.1937989)
Supplement: Supplementary file 2 [file Table_1.docx]

**Supplemental Table 1. Coding Process for Implementation.**

| **Categories of Recommendations** | **Ratings Scale** | **Overall Complied with Recommendations Scale** |
| --- | --- | --- |
| 1. Establish a consistent morning routine | 0= Category of recommendation not given/ not specified/not applicable | 0= Not specified/Not applicable/No did not comply |
| 2. Increase daylight exposure | 1 = Category of recommendation was given | 1 = Partial/Inconsistent |
| 3. Avoid or limit bedroom use |  | 2 = Yes did comply |
| 4. Increase daytime exercise |  |  |
| 5. Avoid or limit daily caffeine intake |  |  |
| 6. Avoid or limit naps |  |  |
| 7. Establish consistent bedtime routine/evening activities |  |  |
| 8. Changes in bedroom environment |  |  |
| 9. Brief & Boring technique |  |  |
| 10. Co-fading techniques |  |  |
| 11. Incorporate visual schedule/visual supports |  |  |
| 12. “Other” Recommendations |  |  |
