# Supplementary material for: Feasibility and effectiveness of a clinical model of sleep education for children with IDD: a record review
Source: Front Sleep. 2026 Sep 9;5:1937989. doi: 10.3389/frsle.2026.1937989 (PMC13597379; doi:10.3389/frsle.2026.1937989)
Supplement: Supplementary file 3 [file Table_2.docx]

**Supplemental Table 2. Pediatric Sleep and Autism Clinical Global Impressions Scale (Pediatric CGI)**

**Sleep Onset Delay**

| 1. No sleep onset delay | - Falls asleep within 30 minutes on all nights, with rare exceptions |
| --- | --- |
| 2. Borderline or barely having sleep onset delay | - Falls asleep within 30 minutes five or more nights a week |
| 3. Mild to moderate sleep onset delay | - Takes 31-60 minutes to fall asleep on 3-4 nights a week. - Takes more than an hour to fall asleep several times a month but less than once a week |
| 4. Moderate sleep onset delay | - Takes 31-60 minutes to fall asleep on 5-7 nights a week - Takes more than an hour to fall asleep 1-2 nights a week |
| 5. Marked sleep onset delay | - Takes more than an hour but less than 2 hours to fall asleep on most nights. - Takes more than two hours to fall asleep on 1-2 nights a week |
| 6. Severe sleep onset delay | - Takes two hours or more to fall asleep on 3-5 nights a week |
| 7. Among the worst sleep onset delay reported | - Takes two hours or more to fall asleep on 6-7 nights a week |

**Pediatric Sleep CGI – Night Wakings**

| 1. No night wakings | - Child stays asleep on all nights, with rare exceptions |
| --- | --- |
| 2. Borderline or barely having night wakings | - Child awakens parent once or twice a week and returns to sleep within 15 minutes on most occasions |
| 3. Mild to moderate night wakings | - Child awakens parent only once during the night, at least 3 nights a week, and returns to sleep within 15 minutes on most occasions. - Child awakens parent several times during the night, no more than once a week, and returns to sleep within 15 minutes on most occasions. - Child awakens parent only once during the night but is up for at least an hour. This occurs no more than once a week |
| 4. Moderate night wakings | - Child awakens parent only once during the night and returns to sleep within 15 minutes on most occasions, and this occurs every night or almost every night. - Child awakens parent several times during the night, several times a week, and returns to sleep within 15 minutes on most occasions. - Child awakens parent only once during the night but is up for at least an hour (but no more than 2 hours). This occurs 2-3 times a week |
| 5. Marked night wakings | - Child awakens parent several times during the night and returns to sleep within 15 minutes on most occasions. This occurs every night or almost every night. - Child awakens parent only once during the night but is up for at least an hour (but no more than 2 hours). This occurs 4-5 times a week. - Child awakens parent only once during the night but is up for more than 2 hours). This occurs no more than once a week. - Child awakens parent several times during the night and is up for at least an hour each time. This occurs no more than once a week. |
| 6. Severe night wakings | - Child awakens parent only once during the night but is up for at least an hour (but no more than 2 hours). This occurs 6-7 times a week. - Child awakens parent only once during the night but is up for more than 2 hours. This occurs 2-5 times a week. - Child awakens parent several times during the night and is up for at least an hour each time. This occurs 2-5 times a week. |
| 7. Among the worst night waking reported | - Every night or almost every night, the child awakens the parent only once during the night but is up for more than 2 hours. - Every night or almost every night, the child awakens the parent and is up for at least an hour each time. |
